# Bibliographical Notices

**Published:** 1856-04

**Authors:** 


### BIBLIOGRAPHICAL NOTICES.

*Memoirs of the Life and Services of* DANIEL DRAKE, M. D., *Physician, Professor, and Author ; with notices of the early Settlement of Cincinnati and some of its Pioneer Citizens.* By EDWARD D. MANSFIELD, LL. D., author of "American Education," &c. Cincinnati; Applegate & Co. 1855.

Of the American physicians now gone to their long home, Daniel Drake is unquestionably one of the greatest, one of those most worthy of imitation and of lasting renown. A poor Kentucky ploughboy just turned of 15 years, having merely learned to read, write, and cypher a little, is ambitiously destined by a pennyless father to the profession of medicine; is transferred to Cincinnati, then a village of miserable huts; is given as a student of the great science of medicine, or rather put apprentice to a Dr. Goforth, who sets him to rubbing quicksilver and "committing to memory Quincy's Lexicon, Cheselden on the bones and Jones on the muscles, without specimens of the former or plates of the latter."

Here, in the unremitting performance of all the duties of a shop boy, he remained three years, when the kind hearted preceptor took him into medical partnership and gave him his diploma testifying that his attainments were "ample in all branches of the profession," signing it *William Goforth, Surgeon-General of the First Division of Ohio Militia.* Such was the unpromising origin of a physician who has, by a long life of eminent usefulness and virtue, adorned the profession, showing, like the Bœotian Philosopher, that great and exemplary men are sometimes known to arise from states of society very unpropitious to the culture of intellect.

> "Summos posse viros et magna exempla daturos
> Vervecum in patria crassoque in aere nasci."

But there is nothing without an adequate cause. Dr. Mansfield very justly attributes the beginning of Dr. Drake's elevation to the native superiority of his intellect. This, however, was cherished by the perpetual example of pious and careful parents who secluded him from vicious examples; and by making themselves worthy of his love, insured his continual presence and willing assistance in all their humble occupations even to the beginning of his 16th year.

We shall lay before our readers a brief summary of the labors and sufferings of this eminent man, taken entirely from this biography, recommending them at the same time not to read our review, but the book itself, from which we can promise them both entertainment and profit.

Daniel Drake was born at Plainfield, Essex County, New Jersey, Oct. 20th, 1785. His parents moved to Kentucky when he was about two and a half years old, in company with four other families. This little colony purchased 1400 acres in the midst of the ancient forest and divided it in proportion to their means, 38 acres falling to the lot of poor Drake. Six years afterwards he sold this and bought a farm of 200 acres near Mayslick, 12 miles south-west of the present Maysville. This was in the midst of the forest, "every thing was new and every thing had to be done." Drake himself thus describes their new habitation:

"Father's cabin stood on a side hill, and was not underpinned. The lower end was three feet from the ground, and here was the winter shelter of the sheep, furnishing security from both wolves and weather; still, although there was protection from rain and snow, the cold wind was not excluded, and it often became necessary to bring the young lambs into the cabin above, and let them spend the night near the fire. The exercise of this kind of office towards the young and suffering innocents was, perhaps, one cause of my repugnance to eating their flesh for many years afterwards. Sometimes they would leave their dams, and then it would become necessary to feed them on cow's milk; a labor which generally fell to me, and I used to hold their mouths in the buck-eye bowl, till they *learned* how to drink."

Daniel being now between eight and nine years old, his time was divided between his father on the farm and his mother in the house. Solomon says, "train up a child in the way he should go and when he is old he will not depart from it." If Drake was not now trained in a way that favored his operations in after life, he was daily imbibing parental lessons and habits of industry that remained with him to the end of his days.

"Though he was much occupied with the out-door pursuits of the farmer's boy, he had yet much to do inside the house. Till he arrived at fifteen years of age, his mother had no hired help, except in sickness. Kentucky was a slave State, yet his father never owned a slave, partly because he could not afford it, but more because he had an invincible repugnance to it. He would not have accepted a negro as a gift, and been obliged to keep him as a slave. He sometimes hired one, but always gave the negro something for himself.

"As a consequence of not owning slaves, Mrs. Drake, Daniel's mother, was frequently without any 'help' in her household affairs; and as Daniel had reached his twelfth year, a strong boy, he became his mother's chief assistant. Nor was he an unwilling one; for it afforded the largest part of that narrow circle of amusements, which were to be found on a farm in the back woods. These domestic employments were various; there was the gathering and assisting in cooking the 'truck,' or garden-stuff for the dinner; there was butter making, cheese making, soap making, hog killing, and a multitude of little employments belonging both to house and field, in which Daniel was the help, the laborer and the prime minister of both father and mother. Judging from his reminiscences of this portion of his life, he must have been a parallel to Giles, in Bloomfield's Farmer Boy, of whom it was said—

"'There never lacked a job for Giles to do.'"

In his 12th year his field of observations was greatly enlarged and his mind received a new impulse from visiting the Blue Licks, where they obtained their salt. They were in the practice of taking as much hay as two horses could draw twelve miles over a stoney road, and this load they exchanged for a single bushel. Here he was surprised with the sudden appearance of great rocks and evergreens, tasted the salt water, heard of the mammoth bones that lay buried in the mud, and "he could tell his mother and sister of strange things they had never seen." In after life he supposed that these and other rural scenery had cherished in his mind that poetic love of nature for which he was greatly distinguished, and which was his supreme delight through all his travels in the boundless West, to which he might have added, *in all his travels through life itself.*

"In the meantime he got something, but not very much, from what is commonly called education—that which is learned only from reading. His father's library, as may be supposed, was by no means extensive. It consisted, in his own words, of the Bible, Rippon's collection of Hymns, Dilworth's Spelling Book, an Almanac, and the famous History of Montellion—a romance of chivalry. To these were afterwards made considerable additions, as the young Daniel advanced in the scholastics. He got Webster's Spelling Book—at that time quite a novelty—Entick's Dictionary, Scott's Lessons, Æsop's Fables and Franklin's Life. All these, and some others procured by borrowing, were good in their way. If they were not extensive in learning, or tempting to the fancy, they were useful; they contained the elements of knowledge, and gave no false and vicious ideas of society.

"With this small library, with the woods around, and in a log-cabin school house, our youth commenced his education or instruction; that only which the world calls education, and which certainly is one of its essential parts, without which he could not have been an eminent physician, nor have created an interest beyond the family circle, in the minds of others.

"If the library was meagre, so the schools in that backwoods country were scarcely more abundant. His father, however, managed to send him occasionally to school. He says, 'limited as were my attainments, they exceeded those of most boys around me, who knew much less. Still, as I was going to be a doctor, father decided I must have another quarter's schooling. Accordingly, he subscribed again to Master Smith, who kept a log school-house on the banks of the Shannon, in the woods, just two miles north of where he lived. So I began to resume my suspended school studies; but the corn had to be hoed, and seeding time required the wheat field to be harrowed after the sowers, and seed had to be covered with the hoe, near the stumps; and it was indispensable for me to labor with my hands, as well as head. So I had to rise at the dawn of day and work at the field till breakfast time, then eat and start with my dinner in my hand. As the distance was two miles, I had to use feet as well as head and hands, and generally ran most of the way.'"

"Here he acquired all the academic education, small as it was, which, aside from his self-instruction and his professional acquirements, he was able to obtain; and here, according to the received opinions of the world, his education ceased. But was it so with him? Very different from this was his estimate of human culture. He was one of those, as we shall see in pursuing his thread of life, whose education never ceased, and whose labors were never done. All persons, places and seasons, were to him the means and agents of instruction. Thus he continually illustrated, in his own mind and person, that great principle, that nature and society are but educators. All are ministering spirits, which a strong mind converts into instruments of growth and acquisition. Education, in its length and fulness, is but the vestibule, and they who teach it but the servants in that building of various, beautiful and infinite adornment, into which God has invited every one who is willing to inquire of him and his works! So thought Daniel Drake, and so has thought every man who has accomplished much by living long and living well."

"From his parents he received a religious training which was made altogether more effective upon his future character, from the fact that he was a dutiful and affectionate child. He appears to have been, in all his youthful avocations, by the side of father or mother, their chief help and willing auditor. Of his father, he says, he was a Christian gentleman, who, comparatively without education, knew well the duties and courtesies which that character required. His mother was even less educated, but her spiritual knowledge and especially her sense of religious duty, made her the best of teachers. She, as is ever the case with children, was his religious instructor, and to her his grateful mind turned back, after half a century of trials, duties and struggles, as if even beyond the veil of other worlds, he would still commune with the same mild and gentle spirit!

"Of the influence of his mother he thus speaks, after he had described some of the vices of the neighborhood:

"That I was preserved from any active participation in, or contamination from, these associations, to which I can trace up the ruin of many of my companions, ought to fill my heart with gratitude to God. The influences under Him which protected me, were, I think, in part my natural tastes and feelings, but in greater part, the admonition of my parents, and of mother still more, perhaps, than father."

In December, 1800, in the beginning of his 16th year, his father took him to Cincinnati and put him under the tuition of Dr. William Goforth, a New Yorker of some medical education. Drake says "he had the most winning manners, great kindness of heart, told anecdotes and talked fluently, dressed with precision, having his hair powdered carefully in the morning, his hands gloved, and walking out with a gold-headed cane.'"

In this new place our virtuous young man was beset by many temptations.

"Fort Washington was garrisoned by gay officers and loose soldiers. The village around it was filled with as gay society, though not wanting in some persons of serious and religious deportment. The tone of society was military, and the garrison which gave that tone was (as indeed was the whole army immediately after the Revolution) rather distinguished for the vices of gambling and intemperance. Judge Burnet, who was then a lawyer at the bar, mentions General Harrison, (then a lieutenant,) and one other, as the only officers he knew who did not end their life by intemperance. Of gambling he spoke as a common practice at the garrison. There, surrounded with men of all ages, from the young subaltern to the grey-haired veteran and respectable citizen, nearly all of whom thought it a light matter to engage in these fashionable vices, he was neither seduced by their authority or example. It was, perhaps, from his early observation on these vices, that he remained to the end of life not only abstinent from them but hostile, looking with contempt upon their followers and with abhorrence on their effects."

Then after having been subjected to these temptations four years, he could write to his father—"since I have lived here, I defy the town to impeach me with one action derogatory to my honor or reputation."

In the year 1802 some of the works of Dr. Rush found their way to this remote village, "which to the mind of Drake were food of the freshest and most captivating kind." Though the Preceptor was adverse to the methods of Rush, he had the penetration to perceive and the candor to acknowledge the rising worth of his pupil, and he took him into a medical partnership in May 1804. Of the hardships of his practice his Biographer quotes from himself:

"Every physician was then a country practitioner, and often rode twelve or fifteen miles on bridle-paths to some isolated cabin. Occasional rides of twenty or even thirty miles were performed on horseback, on roads which no kind of carriage could travel over. I recollect that my preceptor started early in a freezing night to visit a patient eleven miles in the country. The road was rough, the night dark, and the horse brought for him not (as he thought) gentle; whereupon he dismounted after he got out of the village, and putting the bridle into the hands of the messenger, reached his patient before day on foot The ordinary charge was twenty-five cents a mile, one half being deducted, and the other paid in provender for his horse or produce for his family. These pioneers, moreover, were their own bleeders and cuppers, and practised dentistry not less certainly than physic; charged a quarter of a dollar for extracting a tooth, with an understood deduction if two or more were drawn at the same time. In plugging teeth, tin-foil was used instead of gold-leaf, and had the advantage of not showing so conspicuously. Still further, for the first twelve or fifteen years, every physician was his own apothecary, and ordered little importations of cheap and inferior medicines by the dry goods merchants, once a year, taking care to move in the matter long before they were needed."

A very little money was thus hardly earned and the two-thirds of it never paid; his partner was poor and very improvident, a heavy judgment for medicine bought in Philadelphia now hung over him and execution was threatened; no wonder then that poor Drake wrote to his father—

"I am heartily sick and tired of living in the midst of so much difficulty and embarrassment; and almost wish sometimes I had never engaged in partnership with him, for his medicine is so near gone that we can scarcely make out to practice, even by buying all we are able to buy. Add to this, it gives me great unhappiness to see him in such a deplorable situation. I get but little time to study now-a-days, for I have to act the part of both physician and student, and likewise assist him every day in settling his accounts."

"I have not been able to purchase those two books I was telling you were at a store in town, but may be I shall before I leave this." The struggle with these difficulties imposed its valuable lesson of self-denial, and he adds, in the same letter, "I owe nothing except to Mr. D., and am determined to owe him but little."

In the fall of 1805 he obtained a loan from a Mr. Taylor, whom he always mentioned with gratitude, and went to Philadelphia to attend medical lectures. These he found even more useful than he had expected and he wrote to his father—"I try not to lose a single moment, seeing I have to pay so dear for leave to stay in the city a few months." Our inquisitive youth desired to see a little of the great world into which he had emerged, and therefore he went on Sundays to the worship of various sects and once or twice at most, he incurred the expense of the theatre. His Biographer says—

"In reviewing the period of his student-life in Philadelphia (short as it was in time) I am struck with the fine example it offers of great resolution, self-denial and industry, in struggling for and attaining a worthy object. Partly by his own practice of medicine, while yet a youth; partly by borrowing and partly from his father, yet in straightened circumstances, he was able to obtain the small sum necessary to pay for his expenses and instruction at Philadelphia. When there, he labored like one who was conscious that life was a battle in which success must be won by labor and effort. 'I attend the lectures, and then study till two in the afternoon. After dinner apply myself closely to books; call for candles, and sit up till one, sometimes two in the morning. This is my constant plan of conduct. I only sleep six hours in the twenty-four, and when awake, try never to lose a single moment. I had not money enough to take a ticket at the Hospital library, and therefore had to borrow books. Several of my fellow students, Dr. Dewees and Dr. Barton, were very kind to me in this way.' "

He returned to the West in the spring of 1806 and practiced medicine in his father's neighborhood; but in the spring of 1807 his old Preceptor left Cincinnati, and Drake by previous concert stepped easily into his place. His practice increased rapidly among the best people and he took his stand in the best circle of society as one of its most prominent members. One of these has written—

"I should think it hardly more than a year that I had the great advantage of his close acquaintance. It was to me of infinite advantage; for I owe to his example and conversation, at a critical period, much of the little in my tastes and acquirements that give me any satisfaction with myself. If there were few intimate friends, there was a decided influence upon a circle of young men drawn together by strong sympathy with his leading tastes. This influence led, for example, to the establishment of a debating society, which was maintained with spirit and success."

To the mental competition in this society he no doubt owed much of that ready astuteness in debate which, as we have heard, he showed long afterwards in the Medical Society of Philadelphia.

In the family of Col. Mansfield he met Harriet Sisson, the Colonel's niece, "a person of much native grace, refined taste, ardent temperament, quick intelligence, without a fashionable education; in a word, she was a child of nature rather than of art." As the Doctor's prospects of practice were quite flattering, they were married in the autumn of 1807.

"Dr. and Mrs. Drake were admirably suited to one another in their genial dispositions, their buoyant spirits, their love of nature and their ambitious aspirations. Their married life continued eighteen years, attended with a large share of human vicissitudes, and not a little of trouble and adversity; yet, in the whole period, with a mutual confidence and devotion seldom equaled, so much so as to seem quite remarkable to those who observed it. Mrs. Drake, with quick perceptions of her husband's natural talents, and ambitious for his future distinction, ardently assisted him in all his efforts, and exercised much influence over his future career."

When we remember that seven years had not yet passed since he came to Cincinnati, a friendless, uneducated boy of 15 years, and was set to the rubbing of mercury and committing to memory Quincey's Lexicon, it must be confessed that he had effected much, and that he had good reason for indulging the brightest prospects. But sickness and death intervened to give the happy pair some timely experience in the anxieties and sorrows of a married life. In January, 1809, the Doctor had a long attack of pneumonia, from which he barely escaped; and Mrs. Drake suffered so greatly through sympathy and loss of rest, that it was supposed she never recovered her former health. Shortly after this, they lost their little daughter by that awful death—the death from croup. The following lines to his father are too tender and affecting to be left uncopied:

"She was the life, the soul, the comfort of us all; but her mother—her mother—Oh! her mother, what shall I say of her? To her mother she was the *whole* world, except myself. The very existence of this mother was almost inseparable from the life of that little innocent, and now she is almost inconsolable. Every object in the house recalls some little action, some sweet smile, of that little saint! When she lies down at night; when she awakes in the morning; when she comes down stairs; when she sits down at table, she constantly finds something to remind her of the pleasure which that dear little spirit gave her, and, bursting into tears, recounts the innocent smiles and actions of this child of our love."

This, says his Biographer, "is the language of grief, and all who have felt such pangs, will realize the force of these expressions."

But however Drake may have suffered from his own sickness, from the injured health of his wife, and from the loss of his child, he still preserved the vigorous tenor of his way. His ardent and ever active mind made many excursions beyond the limits of his profession. His poetic love of nature, which had been developed in his rustic education, gave him a taste for natural science, which he gratified in his long rides to visit his country patients. He also engaged in palæological inquiries concerning the antiquities of Cincinnati. He investigated and theorized on the remains of ancient fortifications; an Indian mound or cemetery was opened and he examined the remaining sculls, comparing them with the heads of existing tribes. He made some researches in Botany, whether scientifically or according to what system, it is not said; he made a *Calendarium Floræ* and investigated the medicinal properties of plants. Meteorology he did not neglect, but made observations on the winds, rain, temperature, &c., of Cincinnati; so that in 1810 he was already prepared to publish a large pamphlet entitled "*Notices of Cincinnati, its topography, climate, and diseases.*"

This work being found very useful to travellers and scientific inquirers, he received so many applications therefor, that he soon went about preparing something more elaborate on the same subject; this was his *Picture of Cincinnati.*

But unfortunately, this *picture*, the literary associations, and his increasing practice, were all insufficient food for his greedy mind; therefore we find him in conjunction with his brother Benjamin

"commencing a series of commercial operations, which extended through several years, and in the end were very disastrous. For this kind of business he was not well fitted, both because he did not love money enough to be careful of it, and because, like all other professional men, his time and thoughts were necessarily engaged upon other things. The history of literature and of science proves clearly enough that nothing has proved more unfortunate to men in these walks, than commercial speculations. There is something in the pursuit of money and the pursuit of knowledge, which, if not radically hostile to each other, at least admits of no divided empire. Mammon claims all for his own, and looks with jealousy and hate upon all who raise their eyes above the earth."

In remote places it generally happens for obvious reasons that public works of activity or benevolence fall hard on physicians; hence we now find him busy with the institution of a Lancasterian school for which he was Secretary, and also a library company of which he was President. Upon Drake all these literary and scientific avocations fell particularly grievous, in consequence of his deficient education, which he had not yet been able to fill up; and both himself and his biographer have set forth with what labor and difficulty his *Picture of Cincinnati* was at length completed. He says in a letter—

"But even Mr. M. is not fully prepared to imagine, in what a degree of embarrassment I have been immersed for six months past, unless he was obliged to study the *elements* of the sciences to which they belong, to make a dozen applications for a fact, which might be answered in as many words, to exchange the inkstand and pen for the lancet and gallipot every hour in the day, and above all to confront boldly a succession of pertinacious duns. Without all this, and more, he could not experimentally know to what I have been subjected this summer. In the midst of such difficulties and distractions, greater in proportion, I suspect, than those in which Dr. Johnson composed his folio dictionary, nothing but absolute necessity could have *kept me to the course.*"

Dr. Mansfield says that the work was never very popular at home, but that it circulated largely in the Atlantic States and made the name of Drake widely known and respected. We are glad to see that it has long been an inhabitant of the Philadelphia Library. It would be very interesting to compare the productions of his pen at different periods of his career and see by what gradations he attained to such ease and beauty of style, such fulness of thought and flow of sentiment, as have seldom been equalled by his more learned countrymen.

In October, 1815, he went to Philadelphia for the purpose of attending lectures and attaining the Doctorate. It appears that he was favored by the professors with much attention; and he received the longed for and merited degree of M. D. But this proved to be a sorrowful winter to this child of labor; for Mrs. Drake, whom he had taken with him, was sickly the whole time, and in the spring when he was about to be crowned with his degree, he had the sorrow to hear that his little son, whom they had left with his grand parents, had died as did their other child—the death of croup.

On returning to Cincinnati, it was in May 1816, in his 31st year, he quickly fell into a busy and profitable practice, with a bright future opening upon him. "Sed scilicet ultima semper expectanda dies homini." Evil days were approaching, for his parents had removed to Cincinnati and he opened in partnership with his father a store of "dry goods, hardware and groceries," money having been freely borrowed from the bank, without foresight of the perilous time that was then in its birth. We may easily see that this was done for the comfort of his parents in their years of decay, and his total failure in his filial beneficence is therefore to be the more lamented. How these good parents fared afterwards, what comforts they enjoyed, when and how they died, whether they were soothed in their dying hours by the presence of this good son, an old man who expects soon to die would gladly be informed. Of all these things we are left entirely ignorant, and this we must note as a very great defect in this deeply interesting and beautiful book.

In 1817, the Trustees of the U. of Transylvania established a medical department, and Dr. Drake was appointed professor ot Mat. Med. and Botany. During the summer he suffered severely from dyspepsia, but he palliated the complaint by a strict regimen; and by rest and quiet at Lexington, he entirely recovered his health. With respect to his lectures, his biographer has nothing important to relate. Drake however says he was "dissatisfied with the college," wherefore he resigned in the spring and returned to Cincinnati.

His active mind now found leisure or made leisure, as it always had done, to attend to literary institutions and to every thing that could tend to the prosperity of the rising city or to that of the whole Western country. He lectured on botany and medicine to a small class and began to make a show of establishing a medical college at Cincinnati. This frightened the Lexington gentlemen,—not out of their wits as people say, but into their wits, for they forthwith offered him the first professorship in the University, if he would settle permanently in their town. But this he promptly declined, for he foresaw the future superiority of Cincinnati and its adaptation to a medical college. Nor was he slow in his movements, for he went that same year to the Legislature and dunned them into a charter with a large endowment.

"When the fires of ambition had excited his energies—when he had become one of the most prominent citizens, and acquired a wide and brilliant reputation. The multifarious employments into which he had so zealously entered had, however, already begun to task his powers, and teach him the wearisome exactions of professional and public life. In one of his letters, dated October, 1819, he says: 'I have more than once told you how I regretted that my engagements and pursuits have multiplied so much as to materially interfere with my social relations, and fearfully to abridge the hours which ought to be spent in devotion before the shrine of friendship. *When* I shall extricate myself from these enemies of social enjoyment, I cannot predict. The ties which bind me to the world at large seem every day to increase in strength and numbers. The crowd of mankind with whom I have some direct or indirect concern, thickens around me, and I see but little prospect of more leisure, nor any of retirement and seclusion.' "

Many difficulties and sore disappointments attended the opening of the college. Five professors had been appointed by the Legislature, and Drake was President of the College by law. The dangerous power of expelling and electing each other had been chartered to these men, and this power was not long dormant; for two were expelled and two others elected before the college was opened, by which *concordia discors*, a whole year was lost. Whether the first session passed in harmony, we are not told ; but two of the five withdrew at the close and two others expelled Drake, leaving themselves masters of the college which he had founded ! ! The whole community were shocked with this flagrant impudence, and Drake published the causes of this act of folly and suicide in a pamphlet entitled *The rise and fall of the Medical College of Ohio.* One of the expellers lectured in conjunction with one colleague the following year, " with a handful of pupils and no reputation."

" The time had now come when, for once only in his long citizenship, he hesitated about remaining here. He had given the energy of twenty years quite as much to the public as himself. The growth of the town was in no small degree due to the ' Picture of Cincinnati,' which spread abroad the knowledge of its superior advantages. The taste for literature and science, which had begun to spring up, was chiefly excited and kept alive by himself. The laws instituting the college, the medical school, and the hospital, were procured by him. The endowment, not an inconsiderable one, was mostly due to his exertions. He had talked, written, labored and formed plans for Cincinnati, identifying in his mind (and who would not?) his own fame with the growth and glory of the institutions he founded. But now the scene was changed. His reasonable ambition was charged against him as a fault, or a crime. He was expelled from the Medical College. He was bitterly opposed by many of his own profession—recent comers, who probably owed to his writings any knowledge of Cincinnati. Finally, the commercial disasters of the times swept over the West, and affected him, as they did others, with losses and disappointments. His commercial speculations were a failure ; his purchase of goods in the East, with such high expectations of profit, turned out unfortunately. The business of Isaac Drake & Co.

had to be wound up, and the drug establishment passed into the sole hands of his brother Benjamin. The bright pictures of his imagination faded away, and even his sanguine spirit drooped, as it beheld the ruin of so many fair fabrics, from which he had anticipated so much of advantage to himself and society."

In 1823, when he was now 38 years old, he was elected professor of Mat. Med. in the Univerity of Transylvania, and he removed there with his family. His colleagues were Dudley, Caldwell, Richardson, Brown and Blythe. His labors the first winter were severe but pleasant for him, and time rolled on in real enjoyment; for he had left all his vexations both literary and commercial behind him, and he had found at last, as he had every reason to hope, a safe and pleasant abode.

During Dr. Brown's absence in Europe, Drake delivered 26 lectures for him, and taught something concerning *Sympathy*, which Caldwell, professor of *Institutes*, thought was an interference with his own department and doctrines. They had several meetings and some vehement argumentations in the Medical Society. Drake says, that his opponent "cannonaded" him with his heavy artillery for two hours and twenty minutes, and that he returned this with a fire of two hours and thirty minutes. There was no breach of friendship; all this powder passed off like smoke in the calumet of peace.

Drake now found leisure to turn political writer, and he published a number of papers in the Cincinnati Gazette, in favor of Clay's election to the Presidency; if we may judge from the specimens our author has copied, they were written with very superior force and elegance. Dr. Mansfield says: "they were far beyond the common average of political essays, both in style and thought." We cannot, however, but regret that his precious time was not better employed. For a physician to meddle with acrimonious parties in the State, is an *error loci*, a disease that must often interfere with his professional duties, and sometimes end in mortification.

In the summer of 1825, his 40th year, Drake's greatest possible affliction hung over him with her sable wings. He travelled with his wife for the benefit of her health, but this proved to be her last journey on earth. She contracted the remittent fever in her travels, and reaching Cincinnati it here broke out in its worst form, and soon proved fatal. She appears, from various accounts, to have been a very amiable, lovely, captivating woman; a comfort to her husband in all his difficulties, and the object of his intense affection. "He lost a wife who was loved as few can be loved, and she was now mourned with a grief with which few are mourned." He frequently commemorated the anniversary of her death by a funeral hymn; one of these Dr. Mansfield has copied; it is well worthy, both in thought and versification, of a poet of greater pretensions.

In the autumn, he returned with his children, two girls and one boy, to Lexington, and found 281 students, the largest class that had ever assembled there. Though the school promised so much, though all his associations in Lexington were pleasant and his practice lucrative, he resigned his professorship at the end of the course and returned to Cincinnati. He had entered the Transylvania school with 138 pupils, and he now left it with 281, a number which it never again attained.

On returning to Cincinnati his first business and his main dependance was of course the practice of medicine; but his active spirit required some public avocation. He therefore became joint editor of a medical journal, and sole editor after the first year, changing its title to "*The Western Jour. of the Med. and Phys. Sciences.*" In this same year, 1827, he appeared as principal editor of the *Med. and Phys. Journ.* In this he announced his resolution to prosecute a work concerning which he had long before issued a circular to the physicians of the West, requesting "*such facts and observations as would aid him in the composition of a history of the diseases which occur between the Gulf of Mexico and the Lakes.*" The same year he established an *Eye Infirmary*, with upwards of a hundred respectable men as annual subscribers. Thus we find that in less than a year after his return to his beloved Cincinnati, he was already a busy public benefactor.

Temperance now began to be a subject of general interest, and here Drake took a leading part. He was temperate himself, nay, almost abstemious, and therefore he came to his work with a pure as well as a benevolent mind.

"It was in September, 1827, that a public meeting of citizens was called to meet at the Court-house, and consider the subject of temperance. The meeting was held at three o'clock in the afternoon, and, for those days, was really large and respectable. Many old citizens were present, who were quite familiar with old whiskey, and upon whose cheeks it blossomed forth in purple dyes. To these, and indeed to a great body of people in the West, a temperance speech was a new idea. Dr. Drake was the speaker, and they listened to him with respectful attention, and were by no means opposed to the object. The speech, however, was long. The doctor had arrayed a formidable column of facts. The day was hot, and after he had spoken about an hour without apparently approaching the end, some one, out of regard for the doctor's strength, or by the force of habit, cried out: 'Let us adjourn a while and take a drink!' The meeting did adjourn, and McFarland's tavern being near by, the old soakers refreshed themselves with 'old rye.' The meeting again assembled, the doctor finished his speech, and all went off well. Soon after the temperance societies began to be formed, and the excitement then begun has continued to this day."

"After three years of Journalism and professional practice, Dr. Drake found these insufficient to satisfy the activities of his mind. He still longed for what he knew he was specially qualified—the office of medical teacher in a great school; and he still cherished the idea that such an institution would yet rise in Cincinnati. In this frame of mind he accepted, for a temporary purpose, a professorship in the new school at Philadelphia, acting under the charter of Jefferson College. He seems to have accepted this place with an undefined idea of the result; but, obviously with no intention of removing there. It served the purpose of a post of observation, whence he could survey the ground and choose his position. Arrived at Philadelphia, for the third time, he appeared before them in the new and more eminent character of a successful and distinguished teacher of medicine, where he had once been an humble student. Twenty-five years before, he came there a young man, raw, and almost unlettered, with only his own energy for support, and only his ambition for guide. Having exhausted his last dollar and borrowed of friends, he was obliged to leave the University without a degree. Twelve years after, he returned a successful practitioner, but an earnest student, to earn the degree which he received; and now, he returned again to take rank with the greatest professors, and wear honors hardly won in the several fields of study and conflict. It was honorable to him—it was honorable to the West—it was a fine example to ambitious youth, that he had been able to pursue such a career, and from the dim backwoods, by his own exertions, emerge and come to the front rank of enlightened and learned society."

The above is truly said. But one winter fairly answered all the intentions of his going to Philadelphia; he showed his powers in the great metropolis of medicine; he measured minds with the great men of Philadelphia, and was not found wanting; in the Medical Society he displayed, as I am truly informed, overwhelming powers of argumentation and debate; he extended his profitable acquaintance, and formed the most desirable associations—so that he could return to his people with exultation, *carior sibi with a higher opinion of his own merit.*

On his return, he prevailed on the Trustees of the *Miami University* at Oxford, to establish a medical department at Cincinnati, as the Trustees of Cannonsburg had established their prosperous Jefferson College at Philadelphia. This was to be opened in 1831, with the formidable names of Daniel Drake, Geo. McClellan, John Eberle, and others. But evil was too often crossing the straightforward path of Drake. The friends of the *College of Ohio*, for this was still dragging on a sickly life, fearing the powers of the new institution, very artfully effected a union of the rival schools, and the Miami College was therefore abandoned. A professorship was provided for Drake, but as it was a mere supplement, he resigned it the next year. McClellan and Eberle had remained in the Jefferson.

The cholera soon after invaded Cincinnati, affording Drake a subject of investigation worthy of his powers—

" Now, Dædalus, behold by fate assigned,
A task proportioned to thy mighty mind."

He was an active and philanthropic physician throughout the whole epidemic, but it does not appear that even Drake's brilliancy could enlighten us much on this mysterious disease. He wrote a little book on the subject, but as it revealed nothing, it did not find a wide circulation.

During the three years that followed this pestilence, Drake enjoyed a season of comparative peace and exemption from painful collisions. He pursued his practice, was surgeon of the *Eye Infirmary*, and Editor of the *Journal.* His son Charles, now of St. Louis, was just entering at the bar, and his two daughters were old enough to profit by a community of minds ; he therefore built himself a family house, and established a literary association of distinguished ladies and gentlemen to meet in his parlor for mutual improvement.

" The subjects were always of the suggestive or problematical kind, so that the ideas were fresh, the debate animated, and the utterance of opinions frank and spontaneous. There, in that little circle of ladies and gentlemen, I have heard many of the questions which have since occupied the public mind, talked over with an ability and a fulness of information which is seldom possessed by larger and more authoritative bodies. To the members of that circle, these meetings and discussions were invaluable. They were excited to think deeply of what the many think superficially. They heard the doctor's bell with the pleasure of those who delight in the communion of spirits, and revel in intellectual wealth. Nor was that meeting an unimportant affair; for nothing can be unimportant which directs minds whose influence spreads over a country; and such were here. I do not say what impressions they received ; but I know that persons were assembled there, in pleasant converse, such as seldom meet in one place, and who since, going out into the world, have signalized their names in the annals of letters, science, and benevolence. I shall violate no propriety in naming some of them, for those whom I shall name have been long known to the public. Dr. DRAKE was himself the head of the circle, whose suggestive mind furnished topics for others, and was ever ready to incite their energies and enliven the flagging conversation. General EDWARD KING was another, who, in spirit, manners, and elocution, was a superior man, having the dignity of the old school, with the life of the new. His wife, since Mrs. PETERS, and widely known for her active benevolence, and as the founder of the Philadelphia School of Design, contributed several interesting articles for the circle, and was a most instructive member. Judge JAMES HALL, then editor of the Western Monthly Magazine, whose name is known both in Europe and America, was also there. Professor STOWE, unsurpassed in biblical learning, contributed his share to the conversation. Miss HARRIET BEECHER, now Mrs. STOWE, was just beginning to be known for her literary abilities, and about that time contributed several of her best stories to the press. She was not a ready talker, but when she spoke or wrote, showed both the strength and the humor of her mind. Her sister, Miss CATHARINE BEECHER, so well known for her labors and usefulness in the cause of female education, was a more easy and fluent conversationalist. Indeed, few people have more talent to entertain a company, or keep the ball of conversation going, than Miss Beecher; and she was as willing as she was able. Conspicuous, both in person and manners, was Mrs. CAROLINE LEE HENTZE, whom none saw without admiring. She was what the world calls charming, and though since better known as an authoress, was personally quite remarkable. She, and her highly educated husband, a man on some subjects quite learned, but of such retiring habits as hid him from the public view, were then keeping a popular female seminary in Cincinnati. They were among the most active and interesting members of our coterie. I might name others, whose wit or information contributed to the charms of our intercourse, but I should want the apology which public fame has given to the mention of these."

In 1833, was founded the "*College of Teachers*," the object of which was "to unite and improve teachers and to commend the cause of education to the public." This Institution included some very distinguished ladies and gentlemen. Drake was of course the leader, and he wrote, lectured, and delivered orations. Among these was a discourse on the *Philosophy of Family, School*

*and College Discipline.* This was published in the *Trans. of the College of Teachers* for 1834, and was considered as one of his ablest productions ; a few passages which our author has quoted, afford some brilliant corruscations of eloquence.

Dr. Mansfield gives some account of the distinguished gentlemen and ladies who composed this college, which shows with what noble minds Drake was in constant communion. After having published five or six volumes of *Transactions*, the members became gradually scattered over the earth and the College perished—"*omnia orta occidunt.*" In its meetings, says Dr. Mansfield, "I have heard such discussions as I have neither heard nor read of elsewhere."

"In listening to such men discuss some of the most important points in education, connected in the first place with the metaphysics of the human mind, and then with great social interests to flow from them, I have received a pleasure and a benefit—in vain sought among the ordinary pursuits of human life. The memory of these discussions lingering in my mind calls up the delightful company of friends, and the intellectual brilliance which surrounded them."

Chapter XI is entirely devoted to a review of his labors and travels in the cause of internal improvement; particularly in relation to canals and railroads. Shortly after the Revolutionary war, Dr. Rush predicted in a public discourse, but not without exciting some incredulous smiles, that the time was near when ships would be built at Pittsburg and freighted to Europe. In like manner Drake, in his *Picture of Cincinnati*, published in 1815, had traced the various canal routes which have since been used for connecting the lakes with the Ohio. Such were the telescopic powers of these great men. Drake wrote, travelled and declaimed much in the cause, and it is made almost clear that he was the first to project the great railroad from Charleston to Cincinnati; certain it is, that he was the first to propose it to the world.

It was in the years 1835 and 36 that he expended much of his time and energy in the railroad cause, and yet in these very years he found another business sufficient to occupy almost any other mind. The *Medical* College of Ohio, of which Drake was the founder, and from which he was expelled, had been dragging on a miserable existence, notwithstanding some attempts by the Legislature to reform and sustain it; therefore an attempt was made to establish a medical department in the *Cincinnati College*, an institution which had sprung from the *Lancasterian School* formerly established by Drake. A very strong faculty was procured, including Drake, Gross, and our own beloved James B. Rogers. The professors were cordially united; they labored assiduously; students flocked to their standard; one thing only was wanting to complete success and the happy establishment of Drake in his favorite business, the teaching of medicine. Money could not be procured to repair dilapidated buildings, and procure the various apparatus of a great medical school; the expense therefore falling too heavy on a portion of the faculty, they were compelled to abandon the undertaking, after four years of profuse private expenditure.

Drake was soon after elected a professor in the *Medical Institute* of Louisville, whither he removed about 1840. What success attended his labors here, our author does not say, though he must have remained about nine years. The Trustees having limited the professorial age to 65 years, Drake who was now approaching the interdicted year, very wisely resigned. He was immediately elected to the *Medical College of Ohio*, which he had founded thirty years before.

"The old asperities and controversies had passed away, and with none more entirely and completely than with him. He had forgiven, and he resolved to forget, whatever intervened between him and peace with his fellow-men. He therefore accepted the chair offered him, and for one season lectured, for the last time, within the walls of the Medical College of Ohio."

After he had lectured here one year, the Trustees at Louisville found it convenient to rescind their resolution respecting age and Drake was recalled. But he remained here only two years, for old age was now at his heels and he wished to return to Cincinnati to spend the remainder of his days. "A ceaseless attraction," says Dr. Meigs, "drew him near the spot where rested the remains of her whose image was always present;—that was his home, there he wished to die and be buried." He was then elected professor in the *Medical College of Ohio*, and he engaged with the utmost zeal and alacrity in preparing for the coming course. The faculty was very able and quite harmonious and he had reason to expect some years of active life, for his body was yet vigorous and his mental vivacity undiminished; so that he might hope for a peaceful home in the evening of life, and to see his setting sun go down unclouded—gilding with his last rays his beloved *College of Ohio* whose image, to use his own words, "glowing in the warm and radiant tints of earlier life, was ever in his view."

But, alas for human hopes—death was even now at his door—ωλλα τοι ηδη αγχι παρεστηκεν θανατος. He had hardly begun his course when he found himself much indisposed; and evidently from want of that timely care which physicians like others too often neglect in their own cases, he ceased from his labors on the 5th of Nov. It is consolatory to know that he was attended constantly by his daughter Mrs. Campbell and by his son-in-law A. H. McGuffey, Esq.; to this daughter we are indebted for the particulars of his last sickness and death.

He was confined to his house only nine days, but part of this time was spent in great distress of the brain and nervous system. In the beginning of his sickness he expressed a hope that God would spare him till his "great work" might be accomplished; but towards the last he said that even for this favorite object he had no desire to remain. He found himself incapable of attending effectually to religious concerns, but trusted that he had made a sure covenant with his Maker long before, and the memory of this was now as he said his "unspeakable comfort."

We have now given from this faithful biographer a general view of the life and labors of this excellent man; but the spirit of the book, the vivid life of Drake, we could not transfuse—these must be sought in the book itself. In summing up the character of this object of his admiration and love, Dr. M. has quoted largely from Dr. Gross; so authoritative, so pertinent, and so true are these quotations, that if space permitted we should copy them entire.

The personal appearance of a distinguished man is always expected. He was above the middle stature, rather slender but well proportioned; his face was handsome, and when kindled up by some brilliant thought or fervid emotion, his fine blue eyes "of wondrous powers and penetration, fairly twinkled in their sockets." His countenance always denoted thought and in conversation it beamed with intellect. His language flowed slowly but very correct, his voice was mild and clear, rather below the ordinary key, and his elocution was distinct and easy. He was, as we saw him, totally free from dogmatism, being rather inquisitive and suggestive, with even a semblance of diffidence. His step was quick and elastic. He appeared incapable of fatigue either in body or mind, and all that he did or said denoted energy and firmness.

In his social relations he appeared to have been in a high degree amiable and attractive. He loved his friends and they loved him. His house was the abode of a simple but generous hospitality where very many found a cheerful welcome and particularly all strangers of note. His biographer, who was his fellow traveller for many thousand miles, found that he was endowed with a most happy faculty of introducing himself among strangers, and of winning their benevolence and courtesy. He had therefore acquired what Chesterfield says contributes more than anything else to success in life—"an imposing first appearance."

His habitual industry was carried almost to excess. He seems to have always had present in his mind the old maxim, that "minutes are the gold dust of time," hence he acquired, like Priestley, the art of reading and study at the family fire-side, with his child on one arm while writing with the other.

In the all-important subject of religion, Drake had the advantage of early training. Savage, that unfortunate child of genius, says:—

> No mother's care
> Shielded my infant innocence with prayer,
> No father's guardian hand my youth maintained
> Called forth my virtues and from vice restrained.

The very contrary was the fortunate lot of Drake; his parents were pious and their sincerity obtained the early confidence of their son; so successful then was their teaching, that he was never ensnared by a single vice. He always professed Christianity, and about twelve years before his death, after having thought on the subject a long time, he joined the Episcopal Church in full communion; and as it was not his way to leave anything *undone which ought to be done*, he became a very devoted member. Thinking intensely on any subject always drew forth his pen, hence he wrote for the *Episcopal Recorder* of Philadelphia, a series of able articles with the signature of a *Western Layman*. He was one of the founders of the *Society*

*for the promotion of Evangelical knowledge in the Church*, and he wrote their address to the public; for in whatever was undertaken of public utility, he was always sought for as the leading spirit. He was ever ready, like the good Rush, to acknowledge his faith in religion, of which readiness he gave a most noble example about ten days before he went to receive his reward. It was at a great meeting in honor of Webster that he rose and tenderly exhorted the people to remember that this great man resorted in his last hours to the merits of a Saviour. If the giving of a cup of cold water shall not miss its reward, what will be the reward of this noble exhortation?

He has been accused of being too sensitive to blame and too severe in recrimination; but this was in his earlier days when he was opposed by perverse and weakly minds. He had such large and exalted and generous views of public affairs, that to find public utility thwarted by weakness and folly, was to him no common annoyance. His sense of the duties he owed to the public, was very tender, he did not live for himself alone, hence proceeded all his severity of recrimination. That this never ran into excess, would be to claim for Drake an approach to perfection hardly ever attained.

With respect to his writings, they have been so numerous and on such a variety of subjects that our readers cannot expect of them a particular notice. It would appear that in whatever he or his friends undertook, he was called upon for an oration or an essay, and that he was *semper paratus* always ready to respond. As grinding with the hand-mill in his father's cabin had prepared him for the grinding of quicksilver in Dr. Goforth's shop, so he seems to have been always prepared by some previous training, some fertilization of the mind, for every mental effort; his was not the "*ingenium vano se robore jactans*."

Whether Drake pursued his deficient scholastic studies systematically or merely caught what chance offered or necessity required, we are not informed; nor are we told that he attained his beautiful style by any particular course of reading. Poets have generally excelled in prose, and Drake seems to have viewed all nature with poetic eyes; but whether he was addicted to the reading of poetry, our author has not informed us. But in many of the prose quotations that adorn this biography, there is wit and humor and imagery that denote a high degree of poetic talent, and the poetry quoted sufficiently proves, that he could bring this talent into use. Few medical writers have the lively, spirited, vigorous style of Drake. It is constituted in strong common sense, and is very simple, full of thought and sentiment, without embarrassing involutions. He has furnished a model of medical writing, which it would be well for the young physicians of America to study and imitate; as to us old people, we must be contented with writing as we can, and lament that we neglected Rush and had not Drake.

His mental powers were very great, as shown by his ready adaptation to whatever he undertook or was imposed upon him. Wherever he appeared in the West, he was the leading star, and when he came to Philadelphia, he was not eclipsed. We remember hearing long ago from Dr. George McClellan, and we have just been assured by another most competent witness, that in debate he displayed great powers, and that in the *Phil. Med. Soc.* he bore down all opposition in the majestic march of his intellect. With respect to his "great work" we should be glad to write a few pages, but this article is already too long. It has made the name of Daniel Drake respected in foreign lands, and therefore it ought, as well as the biography of its author, to be found in the hands of every American Physician.

Our old preceptor used to tell his class, that every young man aspiring to eminence ought to read the lives of Linnæus and Priestley; to these let us add the life of Drake. Such men sufficiently prove the truth of Sallust's position, that industry is wanting to man, not additional powers or longer life. *Falso* queritur de natura sua genus humanum, &c.

---

*A Treatise on Fractures in the vicinity of Joints, and on certain Forms of Accidental and Congenital Dislocations.* By ROBERT WM. SMITH, M. D., M. R. I. A., Fellow of the Royal College of Surgeons in Ireland, &c., &c. Dublin: Hodges & Smith, Grafton st. New York: Samuel S. & William Wood, 261 Pearl st. 1854. 8vo pp. 314.

The previous issues of this work have already gained for it an enviable reputation. Since its first appearance in 1847, it has been constantly acquiring a higher position in surgical literature. A collection of a few practical papers on special surgical injuries could hardly be expected to gain a very extended circulation, unless embodying views which are available to the general practitioner, for there are comparatively but few in our profession who are sufficiently interested in the subject of fractures to render it a popular work, and yet no one can examine its pages without the conviction that it is no ordinary book. No one can glance at its carefully executed illustrations without admitting its great superiority over every other book of its kind which has yet appeared ; much less can any one carefully peruse its contents and rise from it without being convinced that it has been the result of great labor, careful investigation and philosophical deduction.

Some of our readers may inquire, whether there is anything new on this subject, whether these injuries are not the same now as formerly, or whether Sir Astley Cooper's work on the subject is not sufficient ; to such a one we would reply by inquiring of him whether he has ever experienced any difficulty in clearly satisfying his own mind as to whether a fracture of the neck of the thigh bone, has occurred within or without the capsule? Perhaps he may remember Sir Astley Cooper's mode of discrimination and feel satisfied with his knowledge upon this subject, based upon such authority, but if he will read the volume before us, he will find Sir Astley's opinions on some points re-reversed, and for reasons which he must admit to be entirely satisfactory. Consequently we consider that such a work should find a place, not only in the library of teachers of surgery and hospital surgeons, but should be in the hands of every one who may be called upon to treat so important an injury as a fracture in the vicinity of a joint.

In connection with the subject of fractures of neck of the femur, he has given the history of a large number of cases, and illustrated the injury by many beautiful wood-cuts. Let the reader study, for instance, Chapter I, on Fractures of the Neck of the Femur, and we are sure that it will amply repay him.

After tabulating 60 cases, he deduces the following conclusions:

"1. A slight degree of shortening, removable by a moderate extension of the limb, indicates a fracture within the capsule.

2. The amount of *immediate* shortening, when the fracture is within the capsule, varies from a quarter of an inch to one inch.

3. The degree of shortening, when the fracture is within the capsule, varies chiefly according to the extent of laceration of the cervical ligament.

4. It also varies according as the fracture is impacted or otherwise.

5. In some cases of intracapsular fractures, the injury is not immediately followed by shortening of the limb.

6. This is generally to be ascribed to the integrity of the cervical ligament.

7. In such cases, shortening may occur suddenly, at a period more or less remote from the receipt of the injury.

8. This sudden shortening of the limb is in general to be ascribed to the accidental laceration of the cervical ligament, previously entire, and is indicative of a fracture within the capsule.

9. The deposition of callus around the fragments is not necessary for the union of the intracapsular fracture.

10. When osseous consolidation occurs in the intracapsular fracture, it is effected by the direct union of the broken surfaces, which are confronted to each other.

11. The osseous union of the intracapsular fracture is most likely to occur when the fracture is of the variety termed "impacted."

12. In the intracapsular fracture, the mode of impaction is different from that which obtains in the extracapsular.

13. The degree of shortening, when the fracture is external to the capsule, and does not remain impacted, varies from one inch to two inches and a half.

14. When a great degree of shortening occurs immediately after the receipt of the injury, we usually find a comminuted fracture external to the capsule.

15. The extracapsular fracture is accompanied by fracture with displacement of one or both trochanters.

16. The extracapsular *impacted* fracture is accompanied by fracture without displacement of one or both trochanters.

17. In such cases, the fracture of the trochanters unites more readily than that of the neck of the bone.

18. The degree of shortening, in the extracapsular impacted fracture, varies from a quarter of an inch to an inch and a half.

19. The exuberant growths of bone met with in these cases have been erroneously considered to be merely for the purpose of supporting the acetabulum and the neck of the femur.

20. The final cause of their formation is the union of the fracture through the posterior intertrochanteric space.

21. The difficulty of producing crepitus, and of restoring the limb to its normal length, are the chief diagnostic signs of the impacted fracture.

22. The position of the foot is influenced principally by the obliquity of the fracture and the relative position of the fragments.

23. Inversion of the foot may occur in any of the varieties of fracture of the neck of the femur.

24. When the foot is inverted, we usually find that either a portion or the entire of the extremity of the lower is placed in front of the superior fragment.

25. In cases of comminuted extracapsular fractures, with fractures and displacement of the trochanters, the foot will generally remain in whatever position it has been accidentally placed; it may be turned either inwards or outwards, or there may be inversion at one time and eversion at another.

26. Severe contusion of the hip-joint, causing paralysis of the muscles which surround the articulation, is liable to be confounded with fracture of the neck of the femur.

27. Severe contusion of the hip-joint may be followed, at a remote period, by shortening of the limb and eversion of the foot.

28. The presence of chronic rheumatic arthritis may not only lead us to suppose that a fracture exists when the bone is entire, but also, when there is no doubt as to the existence of fracture, may render the diagnosis difficult, as to the seat of the injury with respect to the capsule.

29. Severe contusion of the hip-joint, previously the seat of chronic rheumatic arthritis, and the impacted fracture of the neck of the femur, are the two cases most likely to be confounded with each other.

30. Each particular symptom of fracture of the neck of the femur, separately considered, must be looked upon as equivocal; the union of all can alone lead to the formation of a correct opinion as to the nature and seat of the injury."

We copy the above not merely for the purpose of giving our readers some idea of the results of the author's labors upon the first chapter, but in order that they may acquaint themselves with the most recent views upon this subject. We should like to give the conclusions obtained by the author upon all subjects of which he treats, but must content ourselves with a bare enumeration of them.

The second chapter treats of Chronic Rheumatic Arthritis of the Hip Joint, and is illustrated by wood cuts, illustrative of this interesting pathological condition.

In the third chapter the author considers the subject of fractures of the bones of the Fore-arm in the vicinity of the wrist Joint. This fracture has of late years received considerable attention from surgical writers, both in this country and abroad, although it was, until very recently, considered as Désault's luxation of the wrist. Mr. Smith's account of the fracture not only places Mr. Colles' paper published in 1814 upon this subject in its true position, but enters also very fully into the seat and nature of the injury; he asserts, however, that the situation of the fracture is not so high as Colles states it to be, "at about an inch and a half above the carpal extremity of the radius," but that he has never seen it "more than an inch above the carpal end of the bone; in the majority of cases it is not so much." This is unquestionably the true seat of the injury, as is abundantly evidenced by the numerous illustrations with which this chapter abounds.

In the fourth chapter the author gives an account of the Fractures of the Humerus, in the vicinity of the Shoulder Joint, in which several injuries of the part are accurately pointed out, viz: those in which the fracture traverses the bicipetal groove, by which the greater tuberosity is detached from the head of the bone; impacted fractures of the neck of the humerus, of which there are two varieties, extra-capsular and intra-capsular; and separation of the superior epiphysis from the shaft of the humerus.

In the fifth chapter the author considers the subject of fractures of the acromial extremity of the Clavicle. The particular bearings of the conoid and trapezoid ligaments upon fractures in this portion of the bone are commented upon, as well as the deformities which arise when the fracture is between the ligaments, or when it occurs internally or externally to them.

In addition there are chapters upon Dislocations of the Bones of the Foot, Congenital Luxations of the Wrist joint, Shoulder joint, and Dislocations of the Lower Jaw.

In all of these the writer shows not only great familiarity with the subject upon which he writes, but also a discriminative judgment in arriving at his conclusions.

Again we say, that the above work does the author great credit, and that the publishers have brought it out in a style becoming its merits. We are glad to see that it has the imprint of an American publishing house, and hope that it will have a more extensive circulation.

---

*Letters to a Young Physician just entering upon Practice.* By JAMES JACKSON, M. D., Professor Emeritus of the Theory of Practice in the University at Cambridge, &c. Boston: Phillips, Sampson & Co. 1855.

Dr. Jackson, it is well known to most of our readers, is an eminent practitioner of Boston, whose professional attainments and unstained character have obtained their legitimate rewards in the love and respect of every one who knows him. A book from such a man could not fail to be instructive, and its lively and often original descriptions and simple yet graceful style, render it a most agreeable one. The work is, in many respects, *sui generis*, as it not only contains the results of the author's large experience on many points that are interesting to the profession, but discusses, also, the obligatory duties and proper mode of behaviour of the young physician. No parts of it, indeed, will be read with so much interest as those which touch upon these latter points, and were there nothing else to commend in it, they would alone stamp it as the production of a sensible, generous and high-toned mind. They will be chiefly found in the prefatory letter to his friend Dr. J. C. Warren, in the introduction, and in the first letter; and we feel confident that wherever they are read, or by whom, they will be felt to be the words of truth and wisdom.

We regret that our limits will not allow us to make any extracts; we had marked several passages for this purpose, both in the portions we have just mentioned and in the more strictly medical parts of the work, but are so "conditioned in space" that we are obliged to omit them all. We would earnestly urge our readers, however, to read the work themselves; they will find it everything we have described it to be.

---

*An Analytical Compendium of the various Branches of Medical Science, for the use and examination of Students.* By JOHN NEILL, M. D., and J. G. SMITH, M. D., &c. *A new edition revised and improved.* Philadelphia: Blanchard & Lea. 1856.

We are pleased to see that the above work is in its third edition. This demand for it, and what is of equal import, the high character of its authors as teachers, are sufficient guarantees that it is well adapted to the wants of the two classes for whose benefit it was chiefly written—students and their instructors.

The preface states that neither time nor labor have been spared in embodying in it all the recent discoveries and improvements, together with "such alterations as have been suggested by its practical use in the class and examination room."

We recommend it to our readers as the best work of its kind with which we are acquainted.

---

*Contributions to the Physiology of Sight.* By Dr. T. C. Hilgard, of Philadelphia. Cambridge: Metcalf & Co. 1856.

In this paper, extracted from the Proceedings of the Providence Meeting of the American Association for the Advancement of Science, the writer endeavors to show that accommodation for distances by the eye is produced by voluntary muscular action, by which force the eye-ball becomes flattened or elongated in the direction of its axis: the muscles being *relaxed* for great distances and *contracted* for short ones. He also states that the sole office of the retina is to distinguish the form and color of objects, their size and distance being determined by the *mind*, or rather by the memory and experience, resulting from the frequent use of the sense of touch. Some remarks are made upon complementary colors, as well as upon the effect produced by indistinct outlines in painting, the latter illustrated by Raphael's painting of the Madonna di Sisto.

The style in which the paper is written greatly mars its effect. It is, however, well worth reading.

